# Environmental contamination of postmortem blood cultures detected by whole-genome sequencing surveillance

**DOI:** 10.1017/ice.2023.192

**Published:** 2023-12

**Authors:** Alexander J. Sundermann, Marissa Griffith, Vatsala Rangachar Srinivasa, Deena Ereifej, Kady Waggle, Daria Van Tyne, Graham M. Snyder, A. William Pasculle, Tanner Bartholow, Lora Pless, Lee H. Harrison

**Affiliations:** 1 Microbial Genomic Epidemiology Laboratory, Center for Genomic Epidemiology, University of Pittsburgh, Pittsburgh, Pennsylvania; 2 Division of Infectious Diseases, University of Pittsburgh School of Medicine, Pittsburgh, Pennsylvania; 3 Department of Epidemiology, School of Public Health, University of Pittsburgh, Pittsburgh, Pennsylvania; 4 Department of Infection Control and Hospital Epidemiology, UPMC Presbyterian, Pittsburgh, Pennsylvania; 5 Department of Pathology, University of Pittsburgh School of Medicine, Pittsburgh, Pennsylvania; 6 Clinical Microbiology Laboratory, UPMC Presbyterian, Pittsburgh, Pennsylvania

*To the Editor—*Postmortem blood cultures may assist in diagnosing a previously undetermined infection contributing to death or confirming a diagnosed infection prior to death. The collection of the blood culture during autopsy commonly entails aseptically obtaining blood from the heart. The clinical utility of postmortem blood cultures is highly debated given potential for bacterial translocation or contamination.^1^ Whole-genome sequencing (WGS) can identify patient infections that are epidemiologically related, indicating transmission or a common source. At our hospital, we recently initiated a WGS program called Enhanced Detection System for Healthcare-Associated Transmission (EDS-HAT) to enable early detection, investigation, and intervention of hospital outbreaks of bacterial pathogens.^2–5^ Here, we describe a pseudo-outbreak related to postmortem blood cultures that was incidentally detected by EDS-HAT.

## Methods

This study was performed at the University of Pittsburgh Medical Center (UPMC) Presbyterian Hospital, an adult, tertiary-care facility with surrounding affiliated UPMC hospitals. Ethics approval for this study was obtained from the University of Pittsburgh Institutional Review Board, the University of Pittsburgh Committee for Oversight of Research and Clinical Training Involving Decedents, and the UPMC Quality Review Committee.

Beginning in November 2021, isolates from clinical specimens (including postmortem cultures) for select bacterial pathogens were collected and sequenced if the patient had been hospitalized for ≥2 days and/or had had a UPMC exposure in the prior 30 days.^5^ Isolates were sequenced weekly using methods previously described and were examined for genetic relatedness.^5^

We observed autopsy practices in March 2022 and performed environmental cultures of the autopsy suite in May 2022. Cultures were taken using a sterile swab from the sink faucet where a hose connected to the table drain. Swabs were plated on MacConkey Agar containing sorbitol and colistin and were incubated for 48 hours at 35°C.^6^

Data on the number of autopsies and blood cultures performed at UPMC Presbyterian from October 2021 through June 2022 were obtained. Data on possibly contaminated blood cultures, defined as any organism related by WGS without plausible epidemiological links, were merged with unique patient blood-culture isolates and autopsies to calculate an autopsy blood-culture contamination rate.

## Results

From October 2021 through June 2022, we detected 4 clusters of genetically related bacterial species among 13 patients who had undergone autopsy at UPMC Presbyterian (Table 1). Initial investigation revealed that each patient had a brief inpatient stay at 1 of 3 UPMC hospitals and after death had been transported to UPMC Presbyterian for autopsy, suggesting a point source in the autopsy suite. One patient had an antemortem blood culture with *S. marcescens* that was genetically distinct from their postmortem blood culture but was genetically related to a subsequent patient’s postmortem culture. Epidemiological investigation did not find potential transmission routes during hospitalization.

**Table 1.** List of Clustered Isolates From Patients and Environmental Cultures

Patient	Source	Outbreak Day	Organism	Cluster
1	Postmortem blood culture	0	*Serratia marcescens*	1
2	Postmortem blood culture	1	*S. marcescens*	1
3	Postmortem blood culture	12	*S. marcescens*	1
4	Postmortem blood culture	40	*Klebsiella oxytoca*	2
5	Postmortem blood culture	43	*S. marcescens*	1
6	Postmortem blood culture	60	*S. marcescens*	1
7	Antemortem blood culture	90	*S. marcescens*	3
	Postmortem blood culture	92	*S. marcescens*	1
8	Postmortem blood culture	101	*S. marcescens*	1
		101	*K. oxytoca*	2
9	Postmortem blood culture	103	*S. marcescens*	1
		103	*K. oxytoca*	2
10	Postmortem tissue culture	186	*K. oxytoca*	2
		186	*S. marcescens*	3
11	Postmortem blood culture	208	*P. aeruginosa*	4
Environmental	Swab from sink faucet	210	*P. aeruginosa*	4
12	Postmortem blood culture	217	*S. marcescens*	1
13	Postmortem blood culture	224	*S. marcescens*	1

Observation of the suite revealed that the autopsy table was rinsed with tap water using a hose attached to a water source on the table to reduce friction of sliding a decedent from the stretcher to the table. Postmortem blood-culture collection was performed using a sterile syringe inserted in the patient’s inferior vena cava. A swab stick with tincture of benzoin was used for aseptic preparation of blood cultures due to supply chain shortages of povidone iodine. Cultures of the sink faucet swab had overgrowth of *P. aeruginosa*. One sequenced isolate clustered with a patient’s postmortem isolate (cluster 4) collected 2 days prior to the environmental sample, indicating the water as the plausible contamination source.

Between October 2021 and June 2022, 309 autopsies were performed. Among them, 183 (59.2%) had postmortem blood cultures, of which 157 (85.8%) were positive for any bacteria; 18 (11.5%) of these 157 were sequenced according to our inclusion criteria. Among 18 patients with sequenced isolates, 13 (72.2%) were genetically related to at least 1 other isolate. Therefore, the minimum estimate of postmortem blood-culture contamination rate was 7.1% (ie, 13 of 183 postmortem blood cultures performed).

Autopsy staff were educated on the findings and disinfection equipment to use for blood-culture collection, and povidone iodine preparations were supplied to the autopsy suite. At the end of the study period (June 2022), there were no additional cases of contaminated cultures.

## Discussion

In this study, we identified a pseudo-outbreak involving 13 patients who underwent autopsy in the same autopsy suite. The most likely source was a sink used to rinse the autopsy table which was found to harbor an isolate of *Pseudomonas aeruginosa* that was genetically related to a patient’s autopsy blood culture. Education on proper blood-culture disinfection was associated with termination of blood-culture contamination during the study period.

Our study had several limitations. First, we only sampled select bacterial pathogens from patients. However, this limitation only underestimates the true bacterial contamination rate. Second, we started sequencing in November 2021, and it is possible that the pseudo-outbreak began previously. Third, as mentioned above, the contamination rate is likely an underestimate of the true rate. Fourth, we do not know how generalizable our results are to other institutions that perform postmortem blood cultures. Fifth, an environmental source of *S. marcescens* was not identified. However, it is likely *P. aeruginosa* overgrew other organisms present in the sink faucet.

In conclusion, we describe a pseudo-outbreak of contaminated postmortem blood cultures that was detected by WGS surveillance. As WGS surveillance becomes more widespread, this method provides additional opportunity to examine the role of all postmortem cultures. Institutions should examine their practices to ensure diagnostic accuracy and determine the utility of routinely perform postmortem blood cultures.
